# DR2 blocker thioridazine: A promising drug for ovarian cancer therapy

**DOI:** 10.3892/ol.2020.12081

**Published:** 2020-09-09

**Authors:** Min Yong, Tinghe Yu, Si Tian, Shuaibin Liu, Jiao Xu, Jianguo Hu, Lina Hu

Oncol Lett 14: 8171-8177, 2017; DOI: 10.3892/ol.2017.7184

Subsequently to the publication of the above article, and a Corrigendum that has already been published to show corrected versions of Figs. 1 and 2 (DOI: /10.3892/ol.2020.11285; published online on January 9, 2020), the authors have realized that Fig. 4D also featured the incorrect placement of a data panel; essentially, the data panel shown for the A2780, thioridazine experiment was a duplicate of the SKOV3, thioridazine data panel.

The corrected version of Fig. 4, featuring the correct data for the A2780, thioridazine experiment, is shown on the next page. All the authors agree to this Corrigendum. Note that the revisions made to these figures do not adversely affect the results reported in the paper, or the conclusions stated therein. The authors regret that this additional error was not noticed prior to the publication of this article, and offer their apologies to the Editor and to the readers of the Journal.

## Figures and Tables

**Figure 4.**
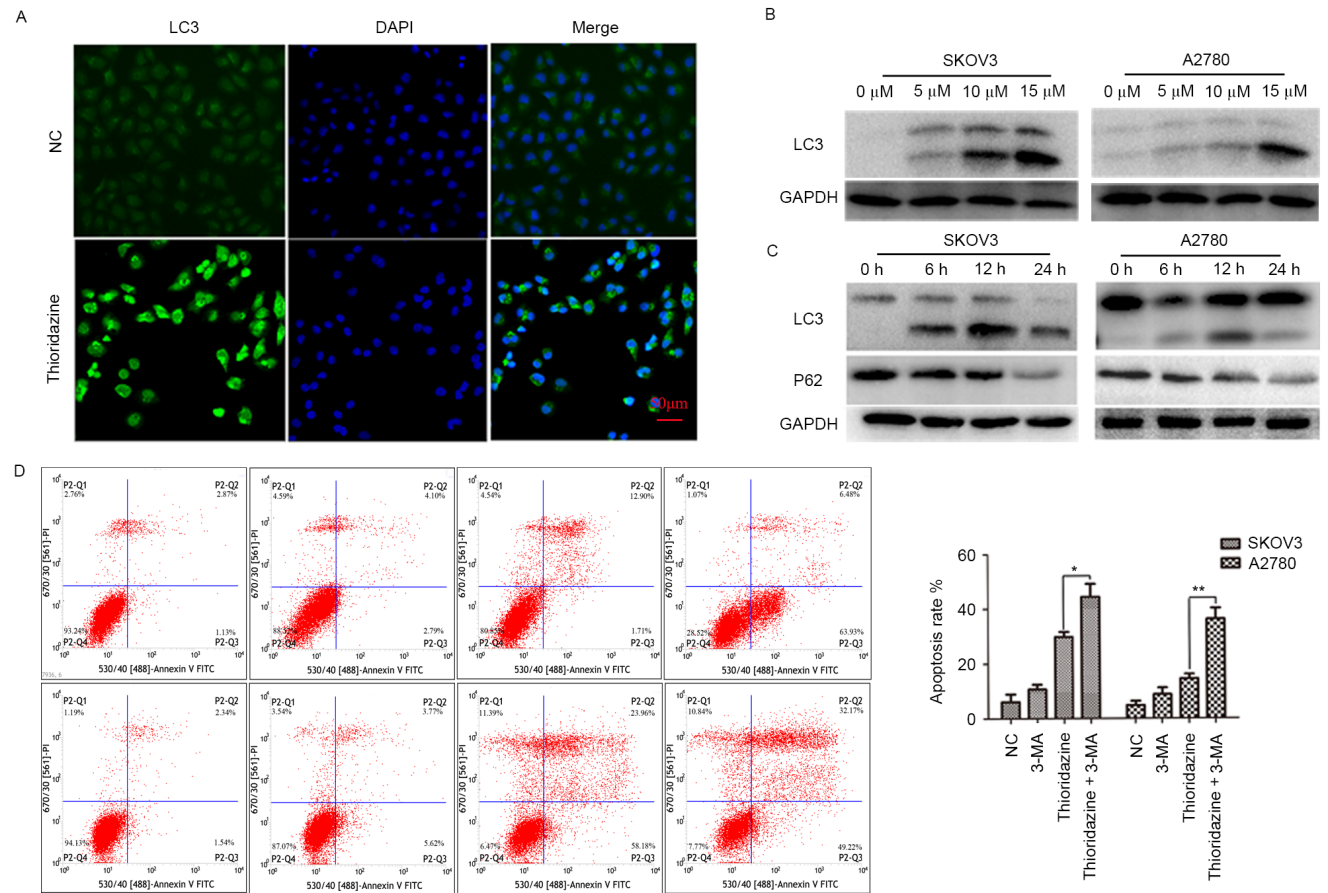
Thioridazine induced autophagy in ovarian cancer cells. (A) LC3 localization following treatment with thioridazine for 12 h (scale bar, 50 μm). (B) Representative western blot analysis demonstrating the expression levels of LC3 following treatment with thioridazine (0, 5, 10 and 15 μM) for 24 h. GAPDH was used as an internal control. (C) Representative western blot analysis demonstrating the expression levels of LC3 and P62 following treatment with 15 μM thioridazine for various lengths of time (0, 6, 12 and 24 h). GAPDH was used as an internal control. (D) Ovarian cancer cells were pre-treated with 5 mM 3-MA for 2 h, and then treated with 15 μM thioridazine for 24 h. Annexin V/PI double staining was used to analyze the effect of thioridazine on ovarian cancer cells. Data are representative of the results of two independent experiments. *P=0.004, **P=0.021 compared with the control group. 3-MA, 3-methyl adenine; NC, negative control; PI, protease inhibitor; LC3, light chain 3.

